# Supplementary material for: Proton Motive Force Inhibitors Are Detrimental to Methicillin-Resistant Staphylococcus aureus Strains
Source: Microbiol Spectr. 2022 Aug 9;10(4):e02024-22. doi: 10.1128/spectrum.02024-22 (PMC9430991; doi:10.1128/spectrum.02024-22)
Supplement: Supplemental file 1 — Supplemental material. Download spectrum.02024-22-s0001.pdf, PDF file, 3.7 MB [file spectrum.02024-22-s0001.pdf]

Supplementary Information

Proton Motive Force Inhibitors are Detrimental to Methicillin-Resistant *Staphylococcus aureus* Strains

Sayed Golam Mohiuddin[1], Sreyashi Ghosh[1], Pouria Kavousi[1], and Mehmet A. Orman[1]*

[1] Department of Chemical and Biomolecular Engineering, University of Houston, Houston, TX, USA

*Correspondence to: S222 Engineering Bldg 1, 4726 Calhoun Rd, Houston, TX 77204, Phone: 713-743-6785, Email: morman@central.uh.edu

SUPPLEMENTARY FIGURES

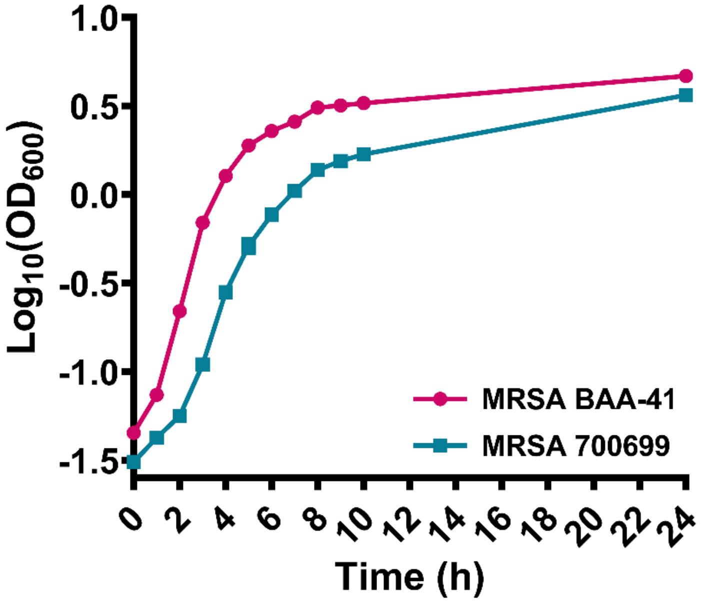

Fig. S1. Growth curves of MRSA BAA-41 and MRSA 7000699 cells. Overnight cultures were diluted (100-fold) in 2 ml fresh Mueller–Hinton broth in 14-ml Falcon tubes and cultured at 37 °C with shaking (250 rpm). At indicated time points, cells were collected for OD_{600} measurements with a plate reader. n = 3. Data points represent mean ± SD.

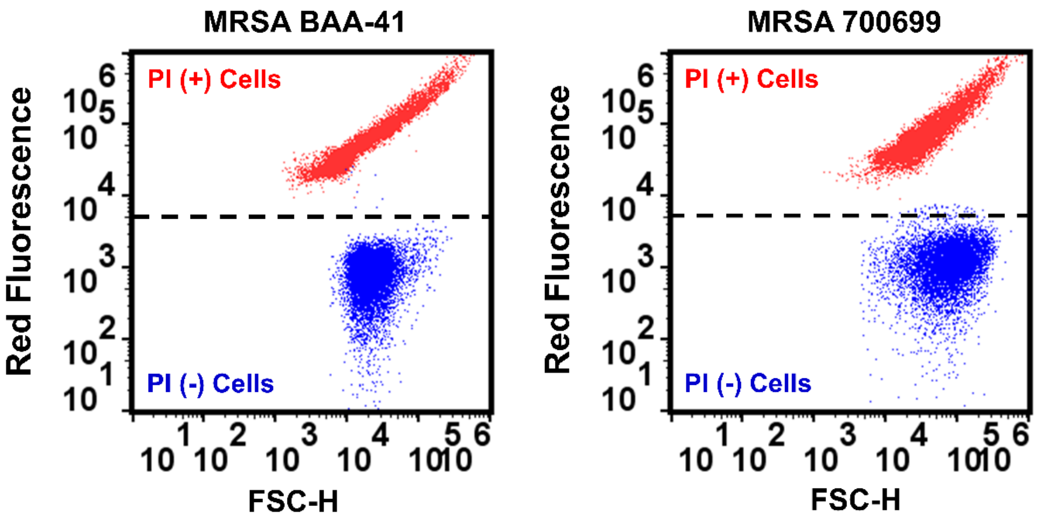

Fig. S2. PI staining of live and dead cells. Live and ethanol-treated (70%, v/v) dead cells were stained with PI (20 µM) dye and analyzed with a flow cytometer to determine the live [PI (-)] and dead cell [PI (+)] subpopulations on the flow cytometry diagram. A representative flow cytometry diagram is provided; all independent biological replicates (n = 3) produced similar results. FSC = forward scatter.

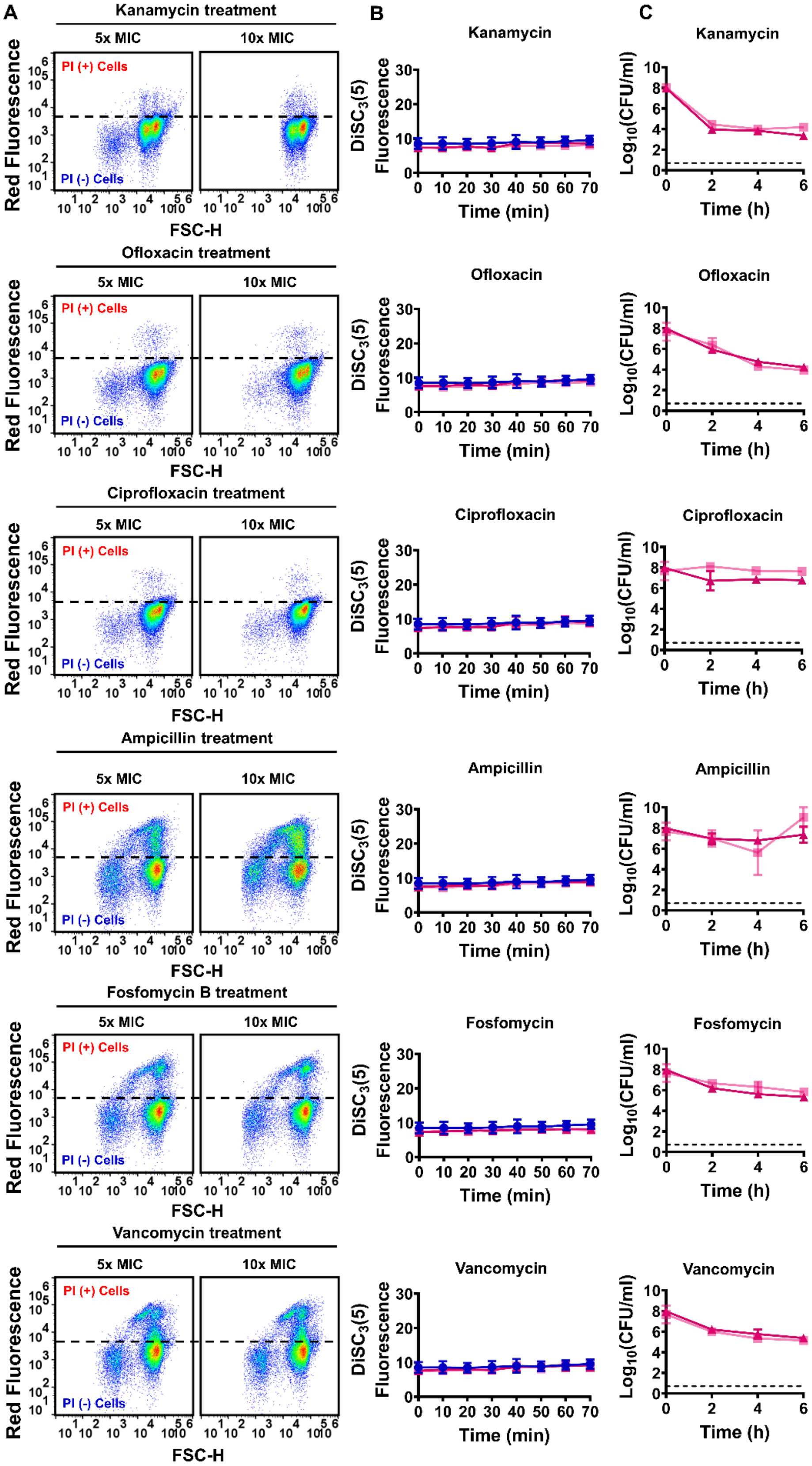
A
Kanamycin treatment
5x MIC
10x MIC
PI (+) Cells
PI (-) Cells
Red Fluorescence
FSC-H
Ofloxacin treatment
Ciprofloxacin treatment
Ampicillin treatment
Fosfomycin B treatment
Vancomycin treatment
B
Kanamycin
Ofloxacin
Ciprofloxacin
Ampicillin
Fosfomycin
Vancomycin
DiSC3(5) Fluorescence
Time (min)
C
Log10(CFU/ml)
Time (h)

Fig. S3. Conventional antibiotics cannot eradicate MRSA BAA-41 cells. Effects of kanamycin (KAN), ofloxacin (OFX), ciprofloxacin (CIP), ampicillin (AMP), fosfomycin (FOS), and vancomycin (VAN) treatments on cell membranes (**A**), PMF (**B**), and cell survival levels (**C)** of MRSA BAA-41 cells were determined as described in **Fig. 1**. A representative flow cytometry diagram is shown here; all independent biological replicates (n = 3) produced similar results. Dashed lines in panel C indicate the limit of detection. Data points represent mean ± SD. Untreated (●), 5× MIC (■), and 10× MIC (▲).

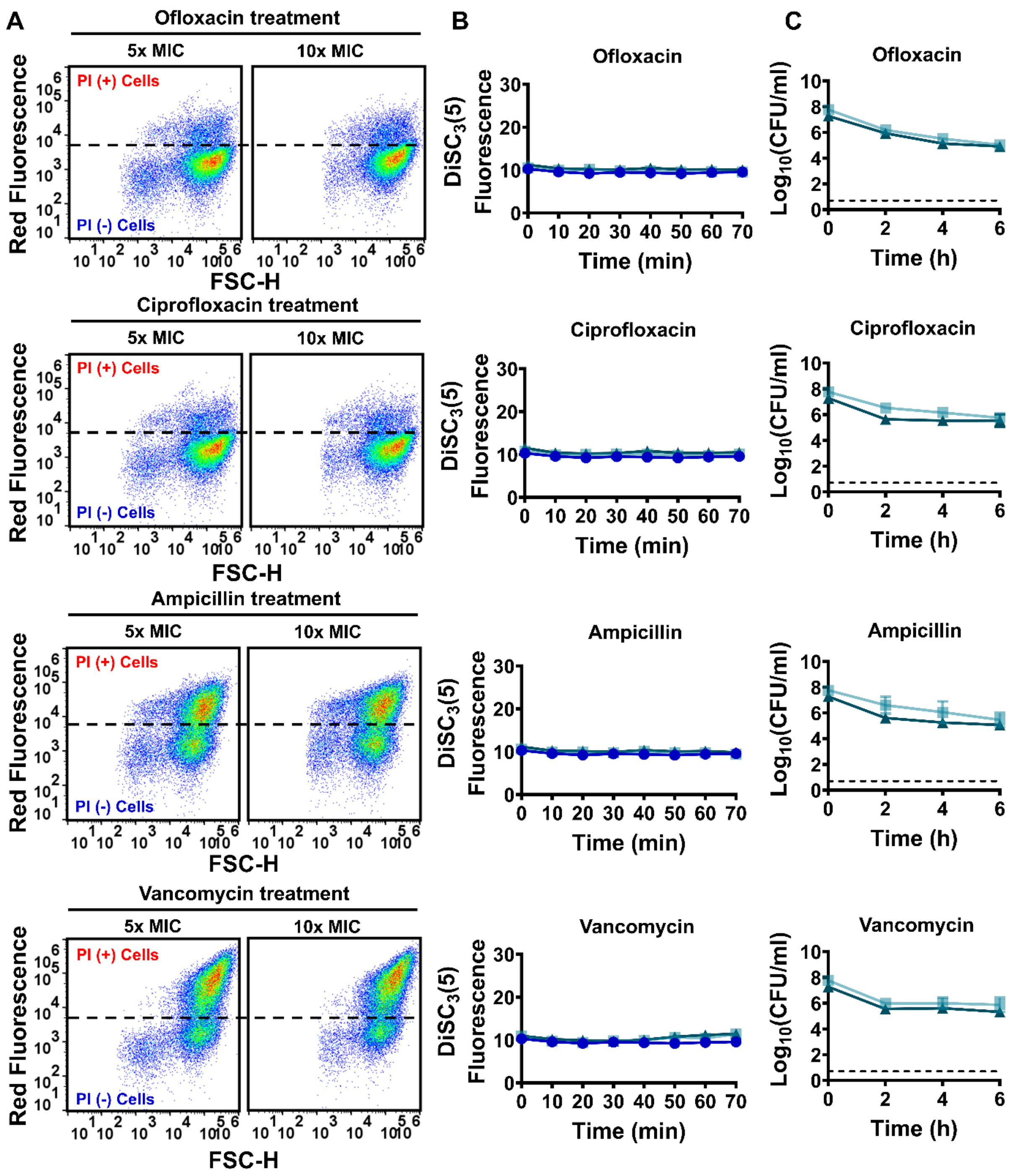

Fig. S4. Conventional antibiotics cannot eradicate MRSA 700699 cells. Effects of ofloxacin (OFX), ciprofloxacin (CIP), ampicillin (AMP), and vancomycin (VAN) treatments on cell membranes (**A**), PMF (**B**), and cell survival levels (**C)** of MRSA 700699 cells were determined as described in **Fig. 1**. A representative flow cytometry diagram is shown here; all independent

biological replicates (n = 3) produced similar results. Dashed lines in panel C indicate the limit of detection. Data points represent mean ± SD. Untreated (●), 5× MIC (■), and 10× MIC (▲).

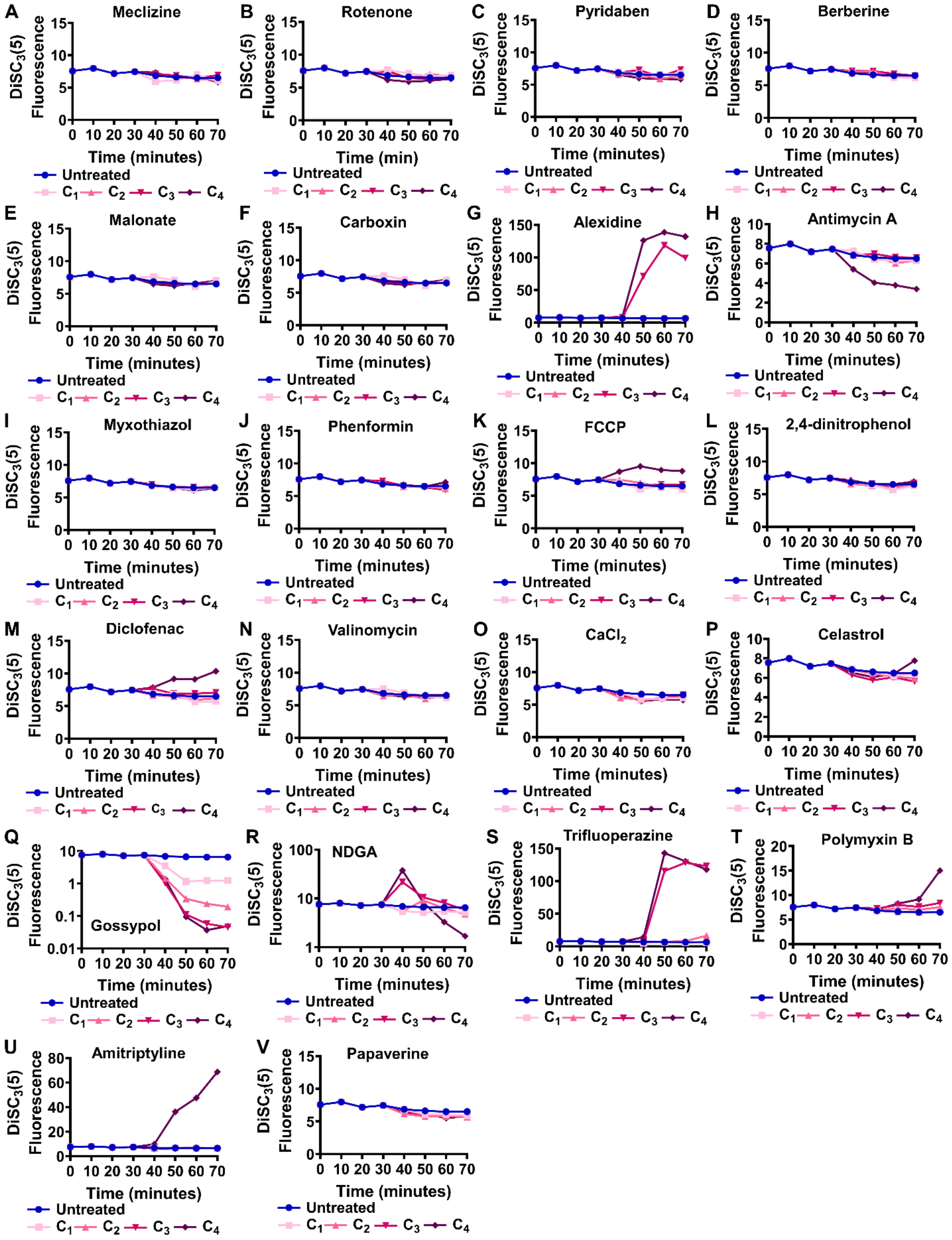
A Meclizine
B Rotenone
C Pyridaben
D Berberine
E Malonate
F Carboxin
G Alexidine
H Antimycin A
I Myxothiazol
J Phenformin
K FCCP
L 2,4-dinitrophenol
M Diclofenac
N Valinomycin
O CaCl2
P Celastrol
Q Gossypol
R NDGA
S Trifluoperazine
T Polymyxin B
U Amitriptyline
V Papaverine
DiSC3(5) Fluorescence
Time (minutes)
Time (min)
0 10 20 30 40 50 60 70
Untreated
C1 C2 C3 C4

Fig. S5. The screening assay to identify PMF inhibitors for strain MRSA BAA-41. (**A–V**) MRSA BAA-41 cells were grown to the exponential phase (OD_{600} of ~0.1) in Mueller–Hinton broth. Exponential-phase cells were then transferred to the $DiSC_3(5)$ assay buffer (50 mM HEPES, 300 mM KCl, and 0.1% glucose) and stained with the $DiSC_3(5)$ probe. When the cells reached an equilibrium state (t = 30 minutes), they were transferred to the MitoPlate I-1 (96-well format) containing drugs at four different concentrations (C_1, C_2, C_3, and C_4). Wells without any drug (A1–A8) were used as controls. The fluorescence levels were measured with a plate reader at designated time points. n = 1.

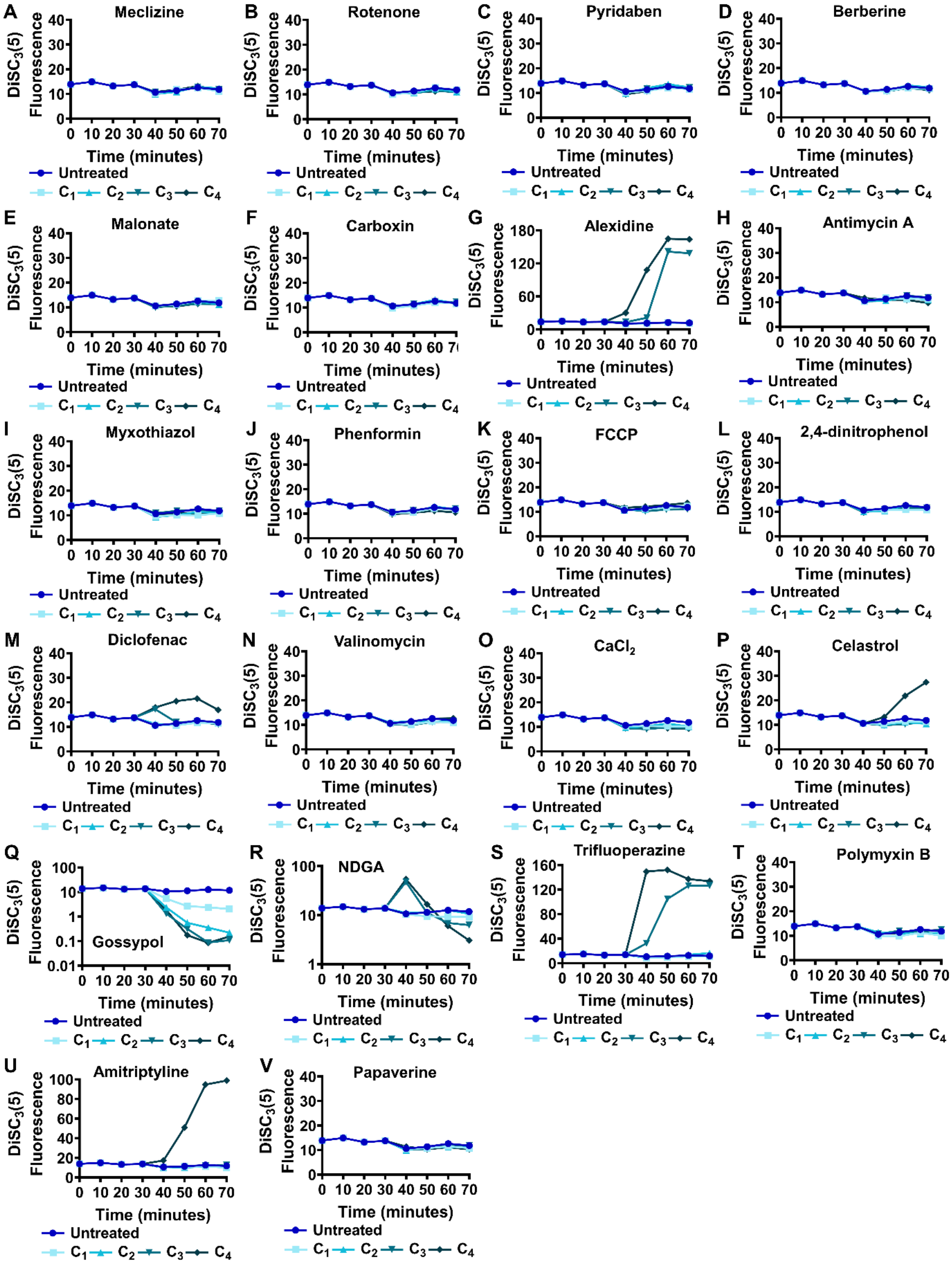
A
Meclizine
B
Rotenone
C
Pyridaben
D
Berberine
E
Malonate
F
Carboxin
G
Alexidine
H
Antimycin A
I
Myxothiazol
J
Phenformin
K
FCCP
L
2,4-dinitrophenol
M
Diclofenac
N
Valinomycin
O
CaCl2
P
Celastrol
Q
Gossypol
R
NDGA
S
Trifluoperazine
T
Polymyxin B
U
Amitriptyline
V
Papaverine
DiSC3(5) Fluorescence
Time (minutes)
Untreated
C1 C2 C3 C4

Fig. S6. The screening assay to identify PMF inhibitors for strain MRSA 700699. (**A–V**) The screening for MRSA 700699 cells was performed as described in **Supplementary Fig. S5**. n = 1.

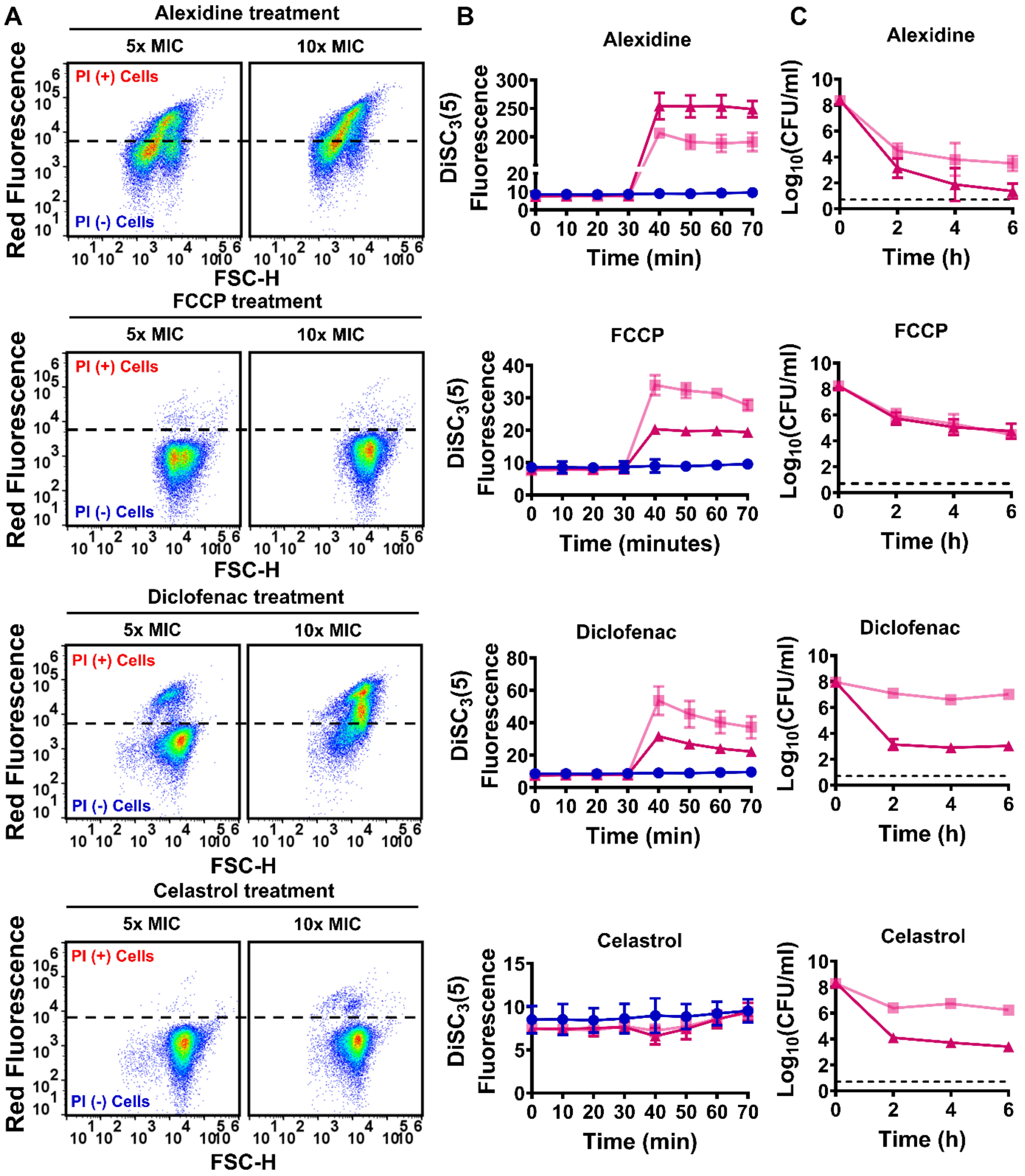

Fig. S7. PMF inhibitors increased membrane permeability, disrupted cellular PMF, and reduced cell survival levels in strain MRSA BAA-41. Effects of alexidine (ALD), FCCP, diclofenac (DCF), and celastrol (CLT) treatments on cell membranes (**A**), PMF (**B**), and cell survival levels (**C**) of MRSA BAA-41 cells were determined as described in **Fig. 1**. A representative flow cytometry diagram is shown here; all independent biological replicates (n = 3)

produced similar results. Dashed lines in panel C indicate the limit of detection. Data points represent mean ± SD. Untreated (●), 5× MIC (■), and 10× MIC (▲).

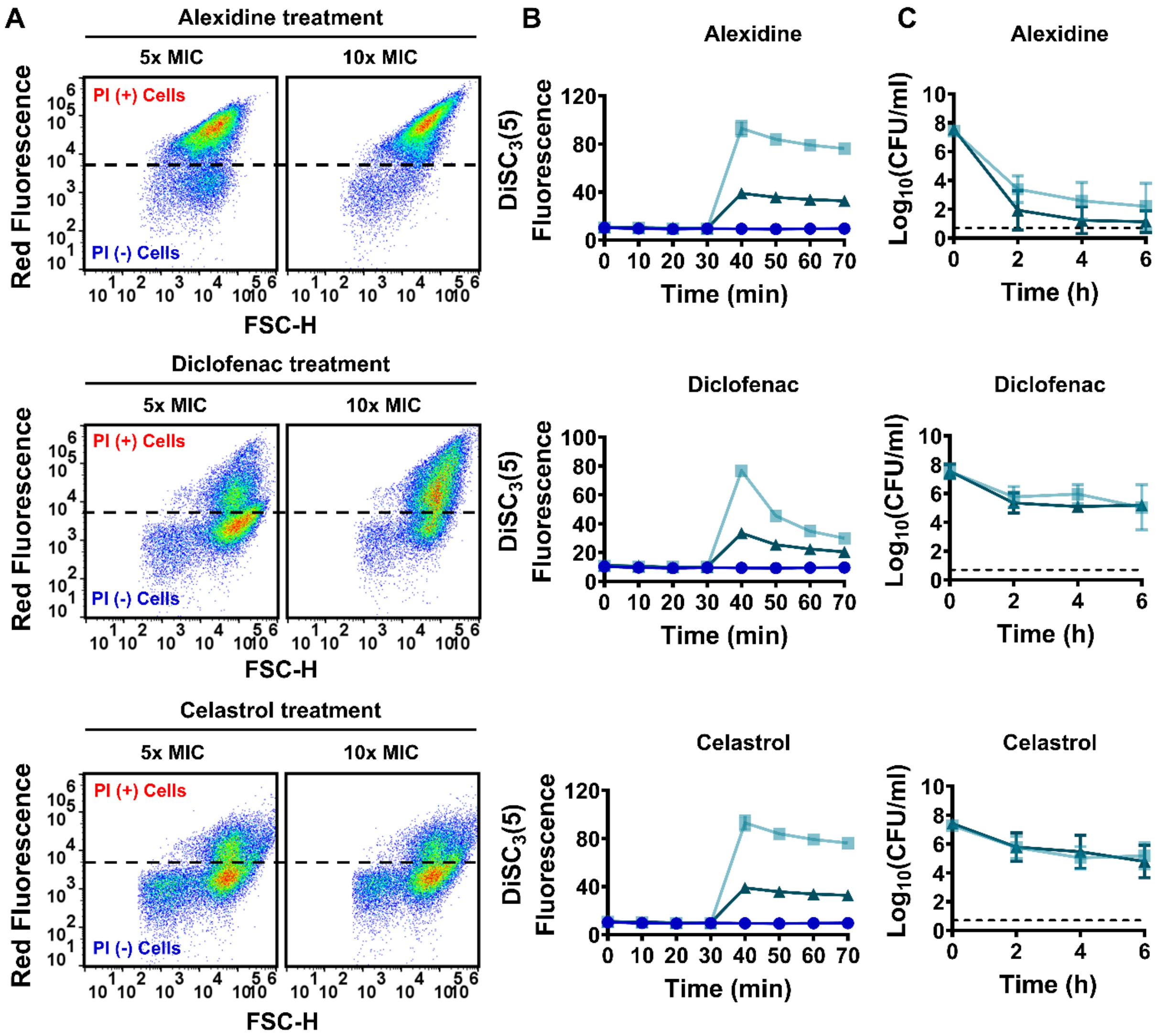

Fig. S8. PMF inhibitors increased membrane permeability, disrupted cellular PMF, and reduced cell survival levels in strain MRSA 700699. Effects of alexidine (ALD), diclofenac (DCF), and celastrol (CLT) treatments on cell membranes (**A**), PMF (**B**), and cell survival levels (**C**) of MRSA 700699 cells were determined as described in **Fig. 1**. A representative flow cytometry diagram is shown here; all independent biological replicates (n = 3) produced similar results. Dashed lines in panel C indicate the limit of detection. Data points represent mean ± SD. Untreated (●), 5× MIC (■), and 10× MIC (▲).

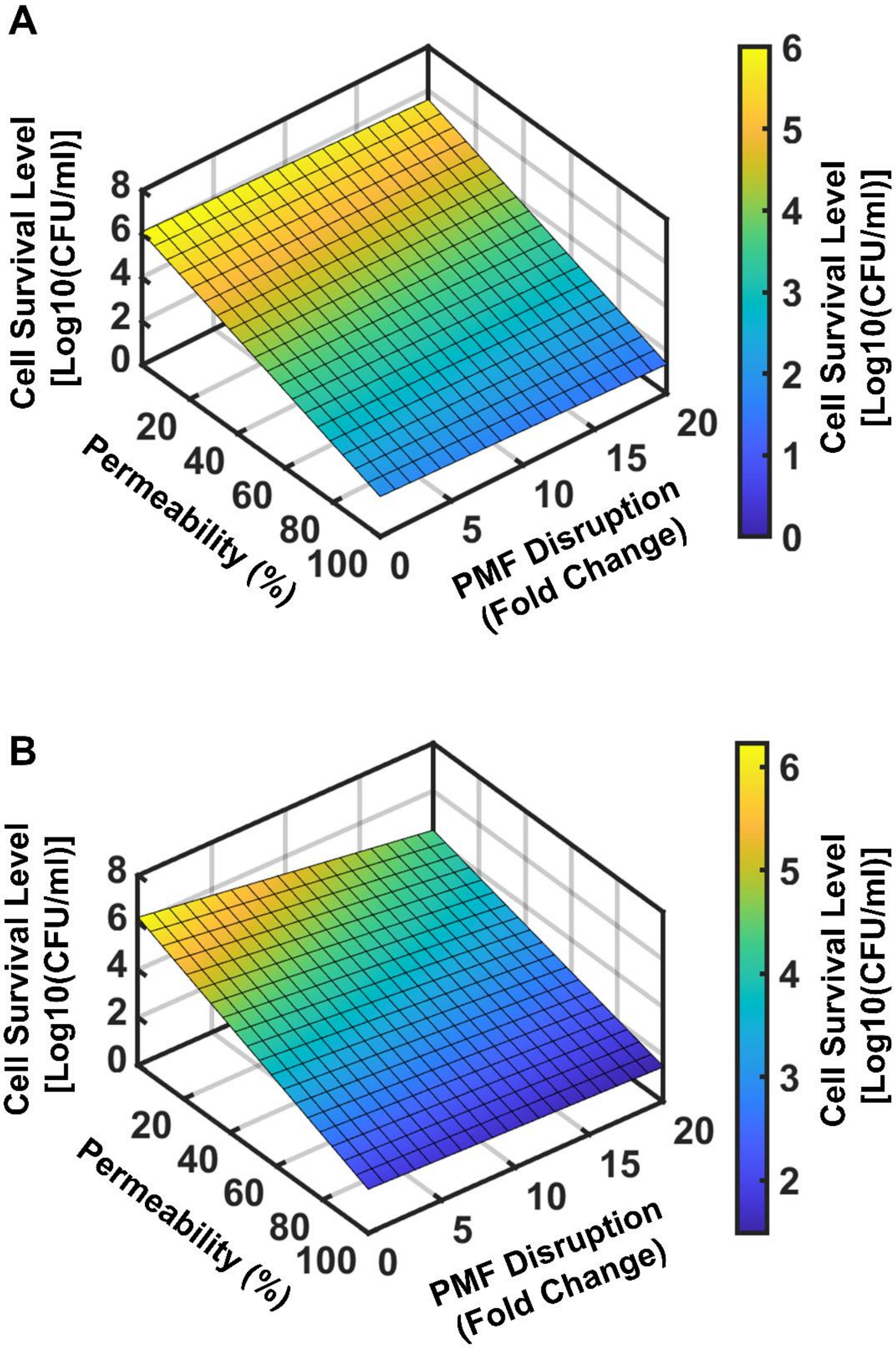

Fig. S9. Simple multivariable regression analysis correlates the disruption of PMF and membrane permeability to cell survival levels. (**A**) Multivariable linear regression analysis without interactions between the independent variables. (**B**) Multivariable linear regression with a two-way interaction between the independent variables. Data obtained from new PMF inhibitors were included. F statistics were used to compare the model equations ($P < 0.01$).

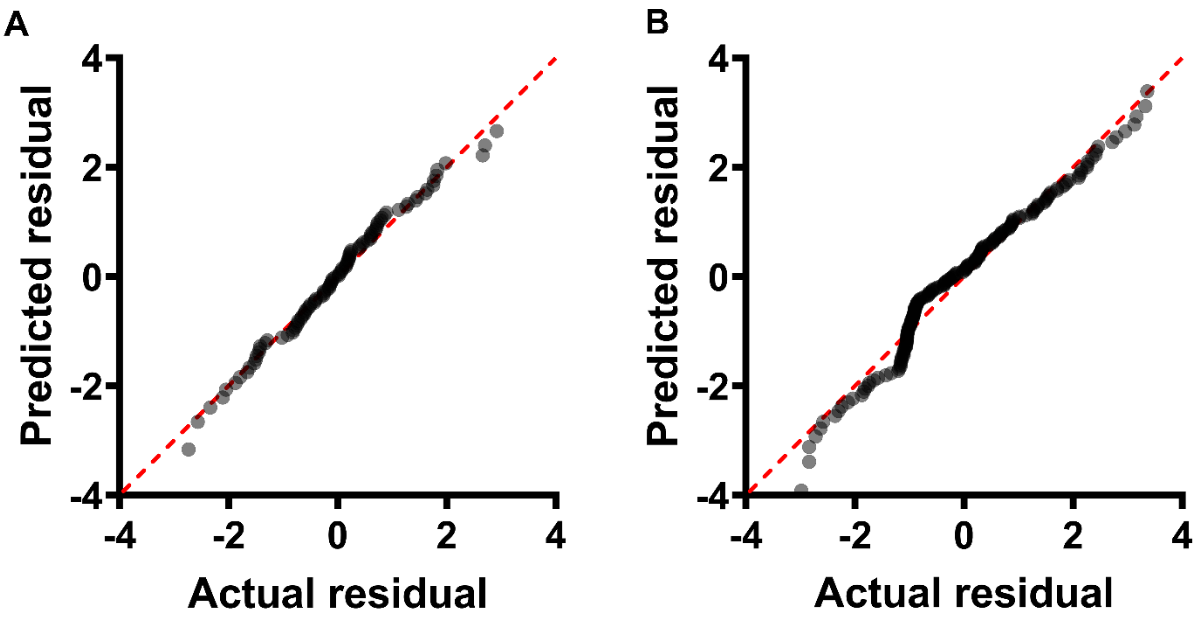

Fig. S10. QQ normality plots. (**A**) QQ plots for the initial dataset of PMF disruption, membrane permeability, and cell survival levels. (**B**) QQ plots for the final data set, including the new PMF inhibitors. These figures were generated for each data set by plotting predicted residuals vs. actual residuals sampled from a Gaussian distribution with a built-in function in GraphPad Prism 9.3.0.

SUPPLEMENTARY TABLES

Table S1: MICs of chemicals.

**A. Known PMF Inhibitors***			
**PMF Inhibitors**	**Bacterial Strains**	**MIC (mM)**	**Clonogenic Survival Assay Concentrations (mM)**
Polymyxin B	MRSA BAA-41	0.03±0.01	0.15 (5× MIC)<br>0.3 (10× MIC)
	MRSA 700699	0.03±0.01	0.15 (5× MIC)<br>0.3 (10× MIC)
CCCP	MRSA BAA-41	0.006±0.002	0.03 (5× MIC)<br>0.06 (10× MIC)
	MRSA 700699	0.006±0.002	0.03 (5× MIC)<br>0.06 (10× MIC)
Thioridazine	MRSA BAA-41	0.0375±0.025	0.1875 (5× MIC)<br>0.375 (10× MIC)
	MRSA 700699	0.023±0.008	0.115 (5× MIC)<br>0.23 (10× MIC)

*A two-fold macro-dilution method was used to determine the MICs.

**B. Conventional Antibiotics***			
**Antibiotics**	**Bacterial Strains**	**MIC (µg/ml)**	**Clonogenic Survival Assay Concentrations (µg/ml)**
Kanamycin	MRSA BAA-41	2.25±0.75	11.25 (5× MIC)<br>22.5 (10× MIC)
	MRSA 700699	>256	N/A
Ofloxacin	MRSA BAA-41	12±4	60 (5× MIC)<br>120 (10× MIC)
	MRSA 700699	16±8	80 (5× MIC)<br>160 (10× MIC)
Ciprofloxacin	MRSA BAA-41	15±9	75 (5× MIC)<br>150 (10× MIC)
	MRSA 700699	15±9	75 (5× MIC)<br>150 (10× MIC)
Ampicillin	MRSA BAA-41	80±48	400 (5× MIC)<br>800 (10× MIC)
	MRSA 700699	9±1	45 (5× MIC)<br>90 (10× MIC)
Fosfomycin	MRSA BAA-41	3±1	15 (5× MIC)<br>30 (10× MIC)
	MRSA 700699	>256	N/A

Vancomycin	MRSA BAA-41	1.5±0.5	7.5 (5× MIC)<br>15 (10× MIC)
	MRSA 700699	9.5±6.5	47.5 (5× MIC)<br>95 (10× MIC)

*Commercially available Etest strips were used to determine the MICs.

Table S2: PMF disruption and membrane permeability data.

A. Known PMF Inhibitors				
**PMF Inhibitors**	**Bacterial Strains**	**Drug Concentrations (× of MIC)**	**Membrane Permeability (%)**	**PMF Disruption (Fold Change)**
Polymyxin B	MRSA BAA-41	5× MIC	88.53±3.07	5.00± 0.45
		10× MIC	93.37±2.24	6.63± 0.67
	MRSA 700699	5× MIC	71.61±1.89	3.67± 0.35
		10× MIC	77.07±0.76	6.63± 1.53
CCCP	MRSA BAA-41	5× MIC	1.15±0.22	1.22± 0.02
		10× MIC	3.29±0.45	1.07± 0.03
	MRSA 700699	5× MIC	7.27±3.32	1.03± 0.02
		10× MIC	7.68±1.05	1.07± 0.03
Thioridazine	MRSA BAA-41	5× MIC	95.39±0.87	12.93±0.35
		10× MIC	96.90±1.51	11.05±0.13
	MRSA 700699	5× MIC	90.33±5.04	16.87± 0.40
		10× MIC	94.38±2.98	14.42±0.61

B. Conventional Antibiotics				
**Antibiotics**	**Bacterial Strains**	**Drug Concentrations (× of MIC)**	**Membrane Permeability (%)**	**PMF Disruption (Fold Change)**
Kanamycin	MRSA BAA-41	5× MIC	1.79±0.50	1.13±0.03
		10× MIC	2.33±0.56	1.06±0.04
Ofloxacin	MRSA BAA-41	5× MIC	2.83±1.57	1.03±0.02
		10× MIC	6.01±6.16	1.02±0.01
	MRSA 700699	5× MIC	11.94±1.96	1.05±0.03

		10× MIC	13.83±3.24	1.05±0.03
Ciprofloxacin	MRSA BAA-41	5× MIC	2.44±1.78	1.05±0.02
		10× MIC	8.71±11.82	1.08±0.07
	MRSA 700699	5× MIC	12.25±1.85	1.04±0.02
		10× MIC	12.69±5.34	1.09±0.03
Ampicillin	MRSA BAA-41	5× MIC	23.25±8.57	1.07±0.05
		10× MIC	28.68±7.31	1.05±0.03
	MRSA 700699	5× MIC	49.80±7.55	1.03±0.02
		10× MIC	45.69±15.73	1.03±0.02
Fosfomycin	MRSA BAA-41	5× MIC	14.47±3.79	1.15±0.01
		10× MIC	17.43±0.52	1.11±0.02
Vancomycin	MRSA BAA-41	5× MIC	24.47±12.57	1.06±0.03
		10× MIC	29.94±4.26	1.15±0.07
	MRSA 700699	5× MIC	68.27±5.32	1.18±0.03
		10× MIC	71.77±0.89	1.19±0.05

**C. Identified PMF Inhibitors**				
**PMF Inhibitors**	**Bacterial Strains**	**Drug Concentrations (x of MIC)**	**Membrane Permeability (%)**	**PMF Disruption (Fold Change)**
Nordihydroguaiaretic acid	MRSA BAA-41	5× MIC	93.97±2.12	20.42±3.29
		10× MIC	93.75±1.86	14.75±2.69
	MRSA 700699	5× MIC	91.73±4.36	29.50±3.82
		10× MIC	92.92±3.67	17.05±1.09
Gossypol	MRSA BAA-41	5× MIC	85.17±9.24	45.61±4.83
		10× MIC	95.02±1.44	89.63±25.99
	MRSA 700699	5× MIC	95.83±2.26	46.82±8.91
		10× MIC	93.66±3.89	122.11±17.18
Trifluoperazine	MRSA BAA-41	5× MIC	95.13±0.87	15.34±1.05
		10× MIC	96.33±1.97	12.78±0.12
	MRSA 700699	5× MIC	92.50±1.25	15.38±0.30

		10× MIC	94.83±0.72	12.25±0.12
Amitriptyline	MRSA BAA-41	5× MIC	92.60±1.31	20.17±1.00
		10× MIC	93.93±0.31	14.78±0.19
	MRSA 700699	5× MIC	95.43±0.25	18.38±0.40
		10× MIC	93.73±1.42	19.07±0.64
Alexidine	MRSA BAA-41	5× MIC	47.33±6.03	21.09±1.78
		10× MIC	57.97±8.55	27.50±1.62
	MRSA 700699	5× MIC	73.01±9.54	23.28±1.16
		10× MIC	90.72±7.14	26.14±0.76
FCCP	MRSA BAA-41	5× MIC	4.52±3.25	3.06±0.19
		10× MIC	8.02±5.83	2.14±0.17
Diclofenac	MRSA BAA-41	5× MIC	11.92±3.07	4.10±0.75
		10× MIC	47.06±33.36	2.45±0.14
	MRSA 700699	5× MIC	38.08±6.10	3.10±0.04
		10× MIC	78.74±4.34	2.12±0.04
Celastrol	MRSA BAA-41	5× MIC	1.68±0.97	1.07±0.01
		10× MIC	1.66±0.88	1.09±0.06
	MRSA 700699	5× MIC	28.94±11.90	7.91±0.17
		10× MIC	31.26±5.43	3.39±0.28

Table S3: Chemicals in the MitoPlate I-1.

A1<br>Control	A2<br>Control	A3<br>Control	A4<br>Control	A5<br>Control	A6<br>Control	A7<br>Control	A8<br>Control	A9<br>Meclizine<br>C_1	A10<br>Meclizine<br>C_2	A11<br>Meclizine<br>C_3	A12<br>Meclizine<br>C_4
B1<br>Rotenone<br>C_1	B2<br>Rotenone<br>C_2	B3<br>Rotenone<br>C_3	B4<br>Rotenone<br>C_4	B5<br>Pyridaben<br>C_1	B6<br>Pyridaben<br>C_2	B7<br>Pyridaben<br>C_3	B8<br>Pyridaben<br>C_4	B9<br>Berberine<br>C_1	B10<br>Berberine<br>C_2	B11<br>Berberine<br>C_3	B12<br>Berberine<br>C_4
C1<br>Malonate<br>C_1	C2<br>Malonate<br>C_2	C3<br>Malonate<br>C_3	C4<br>Malonate<br>C_4	C5<br>Carboxin<br>C_1	C6<br>Carboxin<br>C_2	C7<br>Carboxin<br>C_3	C8<br>Carboxin<br>C_4	C9<br>Alexidine<br>C_1	C10<br>Alexidine<br>C_2	C11<br>Alexidine<br>C_3	C12<br>Alexidine<br>C_4
D1<br>Antimycin A<br>C_1	D2<br>Antimycin A<br>C_2	D3<br>Antimycin A<br>C_3	D4<br>Antimycin A<br>C_4	D5<br>Myxothiazol<br>C_1	D6<br>Myxothiazol<br>C_2	D7<br>Myxothiazol<br>C_3	D8<br>Myxothiazol<br>C_4	D9<br>Phenformin<br>C_1	D10<br>Phenformin<br>C_2	D11<br>Phenformin<br>C_3	D12<br>Phenformin<br>C_4
E1<br>FCCP<br>C_1	E2<br>FCCP<br>C_2	E3<br>FCCP<br>C_3	E4<br>FCCP<br>C_4	E5<br>2,4-Dinitrophenol<br>C_1	E6<br>2,4-Dinitrophenol<br>C_2	E7<br>2,4-Dinitrophenol<br>C_3	E8<br>2,4-Dinitrophenol<br>C_4	E9<br>Diclofenac<br>C_1	E10<br>Diclofenac<br>C_2	E11<br>Diclofenac<br>C_3	E12<br>Diclofenac<br>C_4
F1<br>Valinomycin<br>C_1	F2<br>Valinomycin<br>C_2	F3<br>Valinomycin<br>C_3	F4<br>Valinomycin<br>C_4	F5<br>$CaCl_2$<br>C_1	F6<br>$CaCl_2$<br>C_2	F7<br>$CaCl_2$<br>C_3	F8<br>$CaCl_2$<br>C_4	F9<br>Celastrol<br>C_1	F10<br>Celastrol<br>C_2	F11<br>Celastrol<br>C_3	F12<br>Celastrol<br>C_4
G1<br>Gossypol<br>C_1	G2<br>Gossypol<br>C_2	G3<br>Gossypol<br>C_3	G4<br>Gossypol<br>C_4	G5<br>Nordihydro-guaiaretic acid<br>C_1	G6<br>Nordihydro-guaiaretic acid<br>C_2	G7<br>Nordihydro-guaiaretic acid<br>C_3	G8<br>Nordihydro-guaiaretic acid<br>C_4	G9<br>Trifluoperazine<br>C_1	G10<br>Trifluoperazine<br>C_2	G11<br>Trifluoperazine<br>C_3	G12<br>Trifluoperazine<br>C_4
H1<br>Polymyxin B<br>C_1	H2<br>Polymyxin B<br>C_2	H3<br>Polymyxin B<br>C_3	H4<br>Polymyxin B<br>C_4	H5<br>Amitriptyline<br>C_1	H6<br>Amitriptyline<br>C_2	H7<br>Amitriptyline<br>C_3	H8<br>Amitriptyline<br>C_4	H9<br>Papaverine<br>C_1	H10<br>Papaverine<br>C_2	H11<br>Papaverine<br>C_3	H12<br>Papaverine<br>C_4

Table S4. Vendor, catalog, and purity information of chemicals.

Chemicals	Purity (%)	Vendor	Catalog Number
Polymyxin B<br>(Potency: 7720.0 IU/mg)	N/A	Fisher Scientific	52-915-00MG
CCCP	>98	Fisher Scientific	04-525-00
Thioridazine hydrochloride	>99	Fisher Scientific	30-705-0
Kanamycin sulfate	>95	Fisher Scientific	AC450811000
Ofloxacin	98	Fisher Scientific	AC455670050
Ciprofloxacin	98	Fisher Scientific	AC449620250
Ampicillin	>95	Fisher Scientific	BP1760-25
Fosfomycin	≥98	Fisher Scientific	F08895G
Vancomycin hydrochloride	92.2	Fisher Scientific	ICN19554001
Nordihydroguaiaretic acid	≥97	Fisher Scientific	D08005G
Gossypol	≥98	Fisher Scientific	AAJ63767MD
Trifluoperazine di-hydrochloride	≥98	Fisher Scientific	T28495G
Amitriptyline hydrochloride	≥98	VWR	50-144-4347
Alexidine	≥98	Fisher Scientific	39-795-0
FCCP	≥98	Fisher Scientific	50-201-0426
Diclofenac	98	Fisher Scientific	AC445250100
Celastrol	≥98	Fisher Scientific	C273725MG

Table S5. Dissolving solvents and stock solution concentrations of chemicals.

Chemicals	Dissolving Solvent	Stock Solution Concentration
Polymyxin B	DI water	10 mg/ml
CCCP	DMSO	0.1 M
Thioridazine hydrochloride	DI water	0.1 M
Kanamycin	DI water	50 mg/ml
Ofloxacin	DI water	5 mg/ml
Ciprofloxacin	DI water	10 mg/ml
Ampicillin	DI water	100 mg/ml
Fosfomycin	DI water	100 mg/ml
Vancomycin	DI water	10 mg/ml
Nordihydroguaiaretic acid	DI water	0.1 M
Gossypol	DI water	0.5 M
Trifluoperazine di-hydrochloride	DI water	0.5 M
Amitriptyline hydrochloride	DI water	1.0 M
Alexidine	DMSO	0.1 M
FCCP	DMSO	0.1 M
Diclofenac	DMSO	0.5 M
Celastrol	DMSO	0.1 M
